# Efficacy and safety of monoclonal antibody against calcitonin gene-related peptide or its receptor for migraine patients with prior preventive treatment failure: a network meta-analysis

**DOI:** 10.1186/s10194-022-01472-2

**Published:** 2022-09-08

**Authors:** Xing Wang, Dingke Wen, Qiang He, Chao You, Lu Ma

**Affiliations:** 1 Department of Neurosurgery, West China Hospital, Sichuan University, No. 37, Guo Xue Xiang, Chengdu, Sichuan 610041 People’s Republic of China; 2 West China Brain Research Centre, Sichuan University, Chengdu, Sichuan 610041 People’s Republic of China

**Keywords:** Migraine, Calcitonin gene-related peptide, Monoclonal antibody, Treatment failure

## Abstract

**Objective:**

The relative effects of monoclonal antibody against calcitonin gene-related peptide (CGRP) or its receptor for adult migraine patients with prior treatment failure remains uncertain. Therefore, this study systematically assessed the comparative effectiveness of different CGRP binding monoclonal antibodies (mAbs) for these patients.

**Methods:**

Several online databases including Ovid MEDILNE, Ovid EMBASE, Cochrane Library, and ClinicalTrials.gov were systematically searched from inception to June 15, 2022. We included randomized clinical trials (RCT) of adult migraine patients with previous treatment failure that assessed any CGRP monoclonal antibody. The primary efficacy outcome was change in monthly migraine days (MMDs), and the primary safety outcome was treatment-emergent adverse events (TEAEs).

**Results:**

Overall, seven studies totaling 3, 052 patients were included. Three-node analysis showed that CGRP mAbs was superior to CGRP receptor mAbs in reducing MMDs (MD: -1.55, 95% CrI: − 2.43 to − 0.44) and improving at least 50% response rates (RR: 1.52, 95% CrI: 1.04 to 2.21). Nine-node analysis showed galcanezumab 240 mg ranked first in reducing MMDs (MD -4.40, 95% CrI − 7.60 to − 1.19) and improving 50% response rates (RR: 4.18, 95% CrI: 2.63 to 6.67). Moreover, treatment with fremanezumab or eptinezumab 300 mg provides a significant advantage over erenumab 140 mg regarding an improved response rate of at least 50%. The analysis did not show difference in incidences of TEAEs and serious adverse events in any of the comparisons.

**Conclusions:**

It appears that CGRP mAbs, especially galcanezumab 240 mg, monthly fremanezumab, and eptinezumab 300 mg, seem to be the best choice for the treatment of migraine patients with previous treatment failures. This finding also calls for future research that examine the associations between these medications in migraine therapy among the same patient group to testify the present findings.

**Supplementary Information:**

The online version contains supplementary material available at 10.1186/s10194-022-01472-2.

## Introduction

Migraine is considered as one of the most important causes of disease-related disability worldwide, contributing to functional impairment as well as substantial social and economic burden [1–3]. Although there are several drugs used for migraine patients, many patients either cannot tolerate the side effects, or do not respond to oral migraine preventive medications. At last, they had to switch, re-initiate or discontinue on-going therapies. Up to 78% of patients with migraine have been reported to experience treatment failure [4, 5]. The burden is even higher for patients who have failed previous migraine preventive treatment [6, 7]. Therefore, developing novel drugs with favorable tolerability and sustained efficacy is urgent needed for migraine patients who failed previous treatments.

Monoclonal antibodies (mAbs) related to the calcitonin gene-related peptide (CGRP) are new therapeutic biologics to prevent migraine [8, 9]. Generally, this class of drugs can be divided into two types, mAbs targeting CGRP including eptinezumab, fremanezumab and galcanezumab, and mAbs targeting CGRP receptor including erenumab [10]. However, in most of the available evidences derived from phase II and phase III RCTs associated with these novel agents, participants who had previously failed prophylactic medication for migraine were excluded [11–13]. This suggests that effects of this class of medicine may be different for patients with treatment-resistant migraine. Moreover, the relative safety and efficacy of these drugs in patients with prior migraine treatment failures have not been investigated in depth due to lack of direct comparison of different types of mAbs against CGRP or its receptor in these patients.

Therefore, we performed a bayesian network meta-analysis to assess safety and efficacy of various types of CGRP related mAbs in migraine patients with prior treatment failures. We also conducted a comprehensive ranking of various medications to determine which medications were the most effective in safely reducing monthly migraine headache days.

## Methods

### Search strategy and guideline

We searched bibliographic databases from inception until June 15, 2022 in several databases including Ovid MEDLINE, Cochrane CENTRAL database, and Ovid EMBASE (Supplementary Table A1). Clinical Research registry portal (ClinicalTrials.gov) and reference lists from the previous systematic review on the same field were also searched to identify additional studies. No language restrictions were adopted. The following MeSH terms and free-text terms such as “migraine”, “calcitonin gene-related peptide binding monoclonal antibody”, “erenumab”, “galcanezumab”, “fremanezumab”, “eptinezumab”, and “randomized controlled trial” were used to identify any eligible publications.

The study was registered in OSF platform (https://osf.io/tr8wh), and implemented following the Cochrane Handbook for Systematic Reviews of Interventions. Reporting the study was conforming to the Preferred Reporting Items for Systematic Reviews and Meta-analyses Extension Statement for network Meta-analyses (PRISMA-NMA) guideline [14].

### Eligible criteria

The eligible criteria were based on the patients, intervention, control, outcome, and study (PICOS) principles. We included randomized controlled trials (RCTs) of adult migraine patients (age ≥ 18 yeas) with previous preventive treatment failures (including studies with an identifiable subset of migraine patients with previous preventive treatment failures). The diagnostic criteria are based on the third version of the International Classification of Headache Disorders (ICHD-III) [15]. We defined intervention as any types of CGRP related mAbs, including mAbs targeting CGRP (galcanezumab, eptinezumab, and fremanezumab) and mAbs targeting CGRP receptors (erenumab). The control group was treatment with placebo or a different type of CGRP related mAb. The primary efficacy outcome was defined as change in monthly migraine days (MMDs). Secondary efficacy outcomes were 50%, 75% response rates (defined as a reduction of the frequency of headache attacks by at least given percentage). The primary safety outcome was defined as treatment-emergent adverse events (TEAEs), and secondary safety outcome was serious adverse events.

Besides, the following studies were excluded, 1) RCTs that compared CGRP related mAb with other pharmacological active agents.

### Selection process and data extraction

After removal of duplicates, two reviewers independently filtered publications that were deemed as ineligible based on reading titles and abstracts. The articles in full text were then reviewed and further excluded based on the inclusion and exclusion criteria. Two reviewers independently accomplished this process. Discrepancies in selection were resolved by a third independent reviewer.

Two authors independently extracted data on study characteristics from the eligible trials using a predesigned table. The following data item were noted, characteristics of study including primary author, year of publication, duration of follow-up; patient characteristics including age, sex, type, and dosage of the therapy agent. Two reviewers independently extracted data from the eligible studies. In case of unclear information and additional information for which no relevant results are reported, the corresponding author of the study would be contacted to request the information. Discrepancies in extraction were resolved by a third independent reviewer.

### Evaluation of risk of Bias and quality of evidence

Risk of bias of each trial was assessed using the Cochrane Statistical Methods Group tool across seven domains [16]. In brief, each domain was judged as low, unclear, or high risk of bias. If each domain is assessed as low risk of bias, then a trial will be rated as having an overall low risk of bias. If necessary data were required, we contacted the corresponding author of the original study for more information.

Besides, the quality of evidence for outcomes would be judged using the Grading of Recommendations Assessment, Development, and Evaluation working group (GRADE) tool across five domains, including publication bias, imprecision, overall risk of bias, indirectness, and inconsistency [17]. The overall quality of evidence of each estimate was rated “high”, “moderate”, “low” or “very low”.

### Statistical analysis

The relevant analyses were a 3-node NMA (CGRP mAbs vs. CGRP receptor mAbs vs. placebo) and a 9-node NMA (eptinezumab 100 mg vs. eptinezumab 300 mg vs. erenumab 70 mg vs. erenumab 140 mg vs. monthly fremanezumab vs. quarterly fremanezumab vs. galcanezumab 120 mg vs. galcanezumab 240 mg vs. placebo). We performed a bayesian network meta-analysis model in R software to incorporate indirect comparisons using the consistency model. The point estimates [mean difference (MD) or relative risk (RR)] and the corresponding 95% credible intervals (CrIs) were obtained from the Markov chain Monte Carlo model. In brief, model was set to be with 40,000 simulated draws after a burn-in of 20,000 iterations. The probability for each treatment agent in each outcome was also estimated to rank the intervention levels in the network meta-analysis. Besides, we used the surface under the cumulative ranking curve (SUCRA) to obtain the probability of ranking from worst to best for each treatment agent. In the case of continuous variables that provided incomplete results, we used the formula recommended by Cochrane Handbook for Systematic Reviews of Interventions [18]. Heterogeneity of treatment effects among included studies was examined using the Cochrane Q test and I^2^statistic. I^2^ of 25%, 50% and 75% represent low, moderate and high heterogeneity. The publication bias was checked via Harbord regression test, Egger regression test, and Begg’s test if ten or more trials were pooled.

Statistical analyses were completed in R (release version 4.0.5) and RevMan (release version 5.4.1; The Cochrane Collaboration) software. Bilateral *P* values less than 0.05 were considered statistically significant.

## Results

### Study characteristics

The initial searching identified 1814 potentially relevant articles (Fig. A1 in the Supplement). At last, seven studies (derived from nine trials) totaling 3052 patients were included in this network meta-analysis [19–25]. One trial evaluated effects between eptinezumab and placebo [19]; three trials evaluated effects between erenumab and placebo [20–22]; one trial evaluated effects between fremanezumab and placebo [23]; and two trials assessed galcanezumab vs. placebo [24, 25]. Participants in each trial ranged from 11 to 890. All the included trials mostly enrolled female patients, and the median of proportion of female was 88%. The median age of the included studies was 45.7 years. Five trials (55.6%) completed follow-up visit until 24 weeks, the others completed follow-up visit until 12 weeks. Most trials (78%) were conducted in multiple countries. Study characteristics was summarized and presented in the Table 1.

**Table 1:** Characteristics of trials included in the systematic review and network meta-analysis

Trial	Trial characteristic	Country (Centers)	No. of patients	Characteristics of patients	Intervention		Control		Primary outcome	Follow up
					Protocol	Age (% female)	Protocol	Age (% female)		
DELIVER 2022	NCT04418765	2 countries (96)	890	two-to-four previous treatment failures	100 mg Eptinezumab; 300 mg Eptinezumab	44.6 (93%); 43.1 (89%)	Placebo	43.8 (88%)	Change in MMDs	24 weeks
LIBERTY 2018	NCT03096834	16 countries (59)	246	two-to-four previous treatment failures	140 mg Erenumab	44·6 (80%)	Placebo	44·2 (82%)	50% response rates	12 weeks
STRIVE 2019	NCT02456740	multiple countries (121)	370	≥1 previous treatment failure	70 mg Erenumab; 140 mg Erenumab	43.1 (79.5%); 41.4 (92.2%)	Placebo	43.9 (84.3%)	Change in MMDs	24 weeks
Hirata 2021	NCT03812224	Japan	117	≥1 previous treatment failure	70 mg Erenumab	44.8 (91.5%)	Placebo	44.9 (89.7%)	Change in MMDs	24 weeks
FOCUS 2019	NCT03308968	14 countries (104)	838	two-to-four previous treatment failures	Monthly Fremanezumab; Quarterly Fremanezumab	45.9 (84%); 45.8 (83%)	Placebo	46.8 (84%)	Change in MMDs	12 weeks
Ailani 2020	NCT02614261	12 countries (116)	98	onabotulinumtoxinA treatment failure	120 mg Galcanezumab; 240 mg Galcanezumab	47.5 (85.7%)	Placebo	47.5 (85.7%)	Change in MMDs	12 weeks
	NCT02614183	USA (90)	11			46.5 (100%)	Placebo	46.5 (100%)	Change in MMDs	24 weeks
	NCT02614196	11 countries (109)	20			44.3 (90%)	Placebo	44.3 (90%)	Change in MMDs	24 weeks
CONQUER 2020	NCT03559257	12 countries (64)	462	two-to-four previous treatment failures	120 mg Galcanezumab	45.9 (84%)	Placebo	45.7 (88%)	Change in MMDs	12 weeks

*MMDs* Monthly migraine days; *USA* United States of America

### Efficacy outcomes

**Fig. 1:**
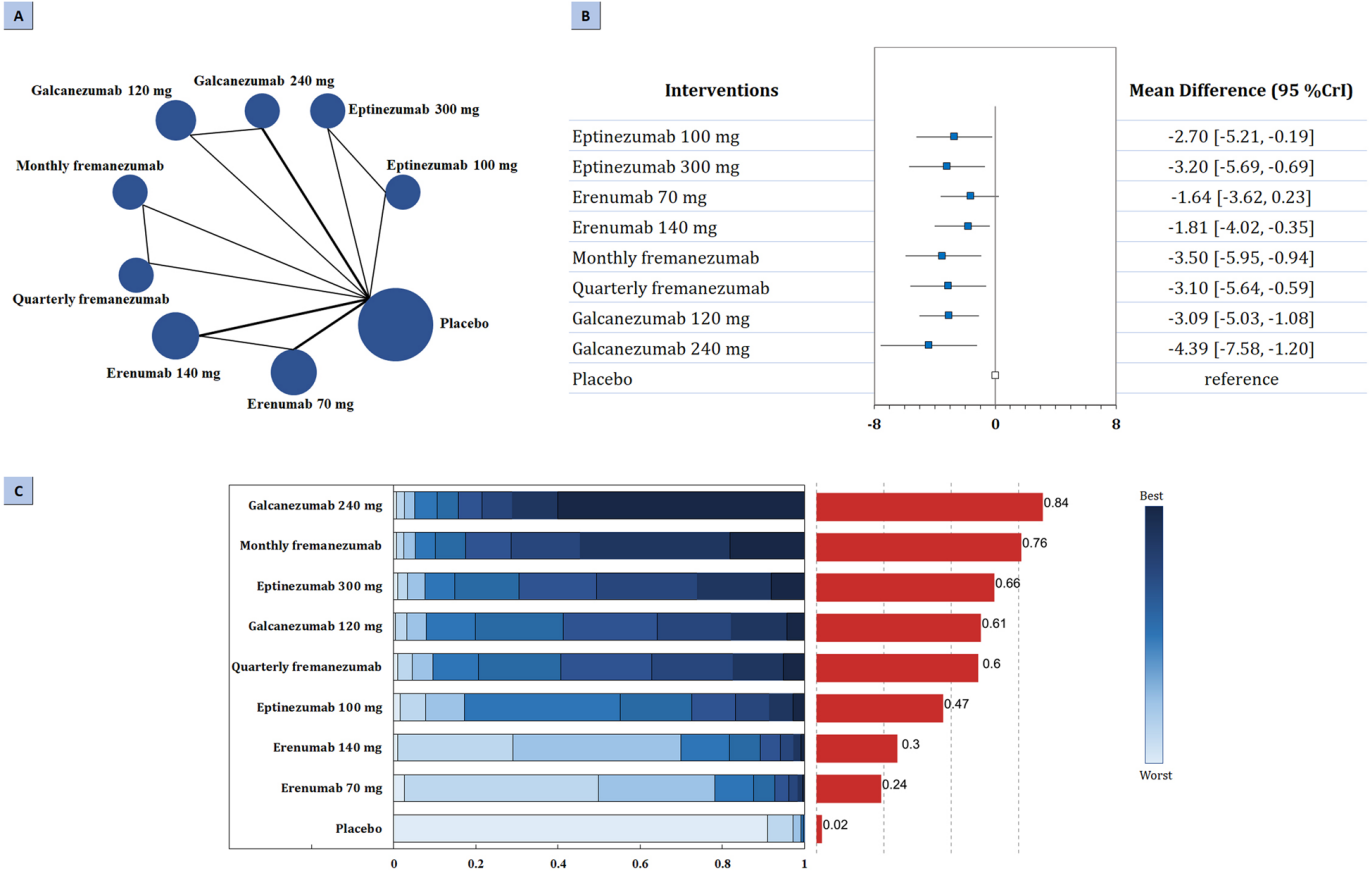
Summary of the primary efficacy outcome. **A** Network plot of change in MMDs. The width of the lines is proportional to the number of studies comparing every pair of treatments, and the size of each circle is proportional to the number of participants. **B** The forest plot shows the risk ratio (RR) and credible interval (CrI). **C** Ranking probabilities graph (blue bars) of each treatment agent. The SUCRA values (red bars) for each treatment are as follows: 84% for galcanezumab 240 mg; 76% for monthly fremanezumab; 66% for eptinezumab 300 mg; 61% for galcanezumab 120 mg; 60% for quarterly fremanezumab; 47% for eptinezumab 100 mg; 30% for erenumab 140 mg; 24% for erenumab 70 mg; 2% for placebo. MMDs: monthly migraine days; SUCRA: surface under the cumulative ranking curve

Regarding primary efficacy outcome, the network of comparison of different types of CGRP related mAbs was reported in all eligible trials totaling 3052 participants (Fig. 1A). According to the three-node analysis (Table 2), both CGRP mAbs (MD: -3.29, 95% CrI: − 3.97 to − 2.76) and CGRP receptor mAbs (MD: -1.74, 95% CrI: − 2.72 to − 1.11) resulted in greater reduction in mean monthly migraine days than placebo. CGRP mAbs also showed better efficacy than CGRP receptor mAbs in reducing MMDs (MD: -1.55, 95% CrI: − 2.43 to − 0.44). Nine-node analysis (Fig. 1B-C) showed that galcanezumab 240 mg had the highest probability of rating first to reduce MMDs (MD -4.40, 95% CrI − 7.60 to − 1.19, SUCRA 0.84), followed by monthly fremanezumab (MD -3.50, 95% CrI − 6.00 to − 0.98, SUCRA 0.76), eptinezumab 300 mg (MD -3.20, 95% CrI − 5.72 to − 0.70, SUCRA 0.66), galcanezumab 120 mg (MD -3.09, 95% CrI − 5.05 to − 1.07, SUCRA 0.61), quarterly fremanezumab (MD -3.11, 95% CrI − 5.60 to − 0.54, SUCRA 0.60), eptinezumab 100 mg (MD -2.70, 95% CrI − 5.15 to − 0.20, SUCRA 0.47), erenumab 140 mg (MD -1.81, 95% CrI − 3.99 to − 0.38, SUCRA 0.30), and erenumab 70 mg (MD -1.64, 95% CrI − 3.63 to 0.18, SUCRA 0.24). Comparisons of drugs with each other did not show significant difference (Fig. A2 in the Supplement).

**Table 2:** Pooled MD/RR and relative CrI derived from three-node network meta-analysis with different treatment regimens in migraine patients with previous preventive treatment failures

Intervention	MD/RR (95% CrI) estimates derived from NMA			SUCRA		
	CGRP mAbs vs. placebo	CGRP receptor mAbs vs. placebo	CGRP mAbs vs. CGRP receptor mAbs	CGRP mAbs	CGRP receptor mAbs	Placebo
Efficacy outcomes						
Change in MMDs	**-3.29 (−3.97, −2.76)**	**-1.74 (−2.72, −1.11)**	**-1.55 (−2.43, −0.44)**	0.99	0.50	0.01
50% response rates	**3.66 (3.01, 4.49)**	**2.40 (1.76, 3.35)**	**1.52 (1.04, 2.21)**	0.99	0.51	0.01
75% response rates	**6.29 (4.07, 10.29)**	**5.38 (2.58, 13.21)**	1.17 (0.43, 2.86)	0.81	0.69	0.01
Safety outcomes						
TEAEs	0.99 (0.88, 1.12)	0.99 (0.87, 1.13)	1.00 (0.84, 1.19)	0.55	0.51	0.44
Serious adverse events	1.31 (0.58, 3.07)	2.24 (0.58, 11.20)	0.58 (0.10, 2.83)	0.50	0.19	0.81

*CrI* Credibility interval; *CGRP* Calcitonin gene-related peptide; *mAbs* Monoclonal antibodies; *MD* Mean difference; *MMDs* Monthly migraine days; *NMA* Network meta-analysis; *RR* Relative risk; *SUCRA* Surface under the cumulative ranking curve; *TEAEs* Treatment­emergent adverse events

**Fig. 2:**
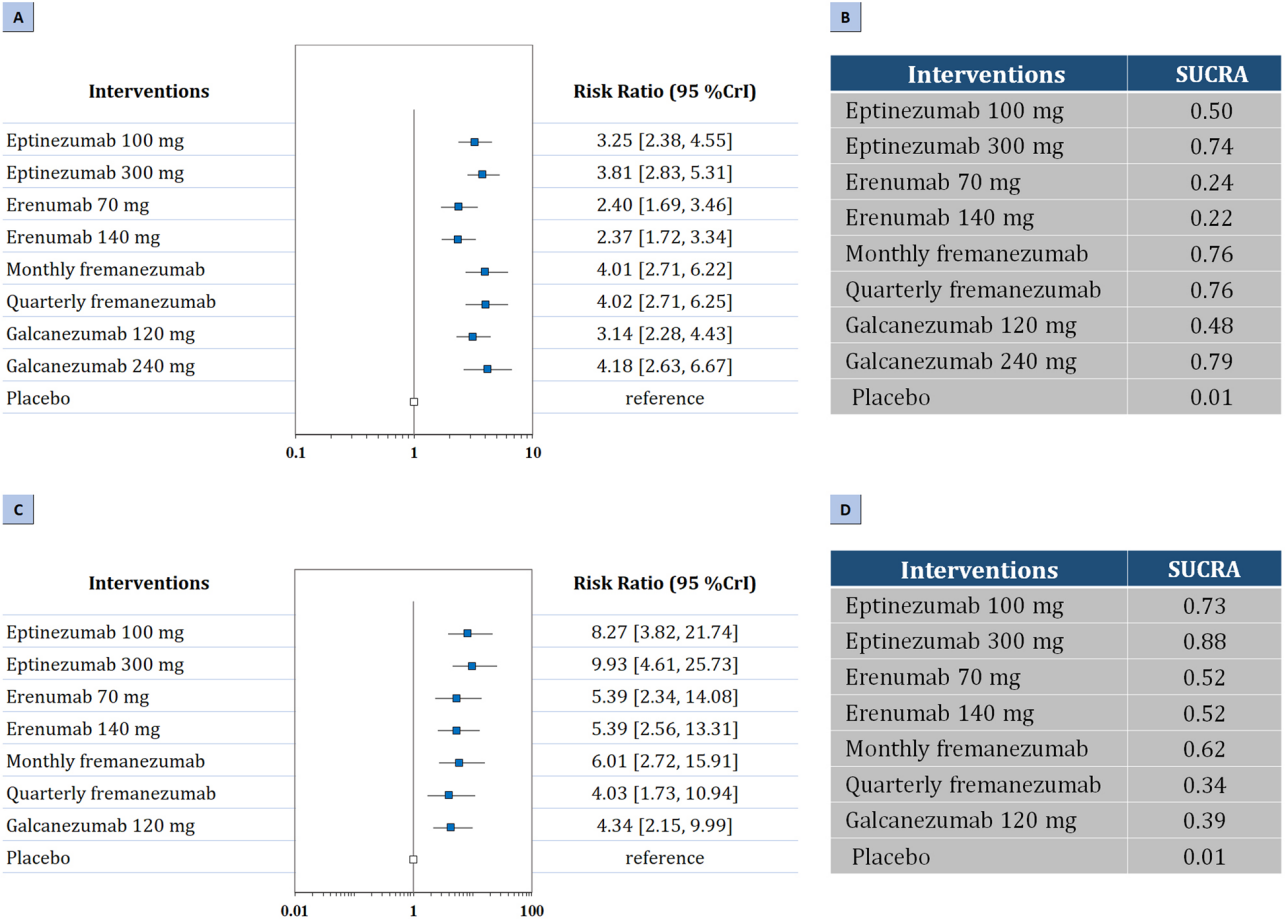
Summary of the secondary efficacy outcomes. Network plot of (**A**) The forest plot for 50% response rates; (**B**) The SUCRA value of each treatment for 50% response rates. (**C**) The forest plot for 75% response rates; (**D**) The SUCRA value of each treatment for 75% response rates. SUCRA: surface under the cumulative ranking curve

Regarding secondary efficacy outcomes, three-node analysis demonstrated that both CGRP mAbs (RR: 3.66, 95% CrI: 3.01 to 4.49) and CGRP receptor mAbs (RR: 2.40, 95% CrI: 1.76 to 3.35) achieved a 50% or greater reduction in the monthly number of migraine days than placebo (Table 2). CGRP mAbs were superior to CGRP receptor mAbs to achieve at least 50% response rates (RR: 1.52, 95% CrI: 1.04 to 2.21). According to nine-node analysis (Fig. 2A-B), galcanezumab 240 mg (RR: 4.18, 95% CrI: 2.63 to 6.67, SUCRA 0.79) had the highest probability of rating first in reducing the frequency of headache attacks by at least 50%, followed by quarterly fremanezumab (RR: 4.02, 95% CrI: 2.71 to 6.25, SUCRA 0.76), monthly fremanezumab (RR: 4.01, 95% CrI: 2.71 to 6.22, SUCRA 0.76), eptinezumab 300 mg (RR: 3.81, 95% CrI: 2.83 to 5.31, SUCRA 0.74), eptinezumab 100 mg (RR: 3.25, 95% CrI: 2.38 to 4.55, SUCRA 0.50), galcanezumab 120 mg (RR: 3.14, 95% CrI: 2.28 to 4.43, SUCRA 0.48), erenumab 70 mg (RR: 2.40, 95% CrI: 1.69 to 3.46, SUCRA 0.24), and erenumab 140 mg (RR: 2.37, 95% CrI: 1.72 to 3.34, SUCRA 0.22). Comparison between different treatment agents showed monthly and quarterly fremanezumab was superior to erenumab 140 mg (RR: 0.59, 95% CrI: 0.34 to 1.00; RR: 0.59, 95% CrI: 0.34 to 1.00, respectivley), and treatment with eptinezumab 300 mg was superior to erenumab 140 mg (RR: 1.61, 95% CrI: 1.02 to 2.56). The results were presented in Fig. A3 in the Supplement.

In addition, three-node analysis demonstrated that both CGRP mAbs (RR:6.29, 95% CrI: 4.07 to 10.29) and CGRP receptor mAbs (RR: 5.38, 95% CrI: 2.58 to 13.21) achieved a 75% or greater reduction in the monthly number of migraine days than placebo. According to nine-node analysis (Fig. 2C-D), eptinezumab 300 mg (RR: 9.93, 95% CrI: 4.61 to 25.73, SUCRA 0.88) had the highest probability of rating first in reducing the frequency of headache attacks by at least 75%, followed by eptinezumab 100 mg (RR: 8.27, 95% CrI: 3.82 to 21.74, SUCRA 0.73), monthly fremanezumab (RR: 6.01, 95% CrI: 2.72 to 15.91, SUCRA 0.62), erenumab 140 mg (RR: 5.39, 95% CrI: 2.56 to 13.31, SUCRA 0.52), erenumab 70 mg (RR: 5.39, 95% CrI: 2.34 to 14.08, SUCRA 0.52), galcanezumab 120 mg (RR: 4.34, 95% CrI: 2.15 to 9.99, SUCRA 0.39), and quarterly fremanezumab (RR: 4.03, 95% CrI: 1.73 to 10.94, SUCRA 0.34). Comparisons of drugs with each other did not show significant difference (Fig. A4 in the Supplement).

### Safety outcomes

**Fig. 3:**
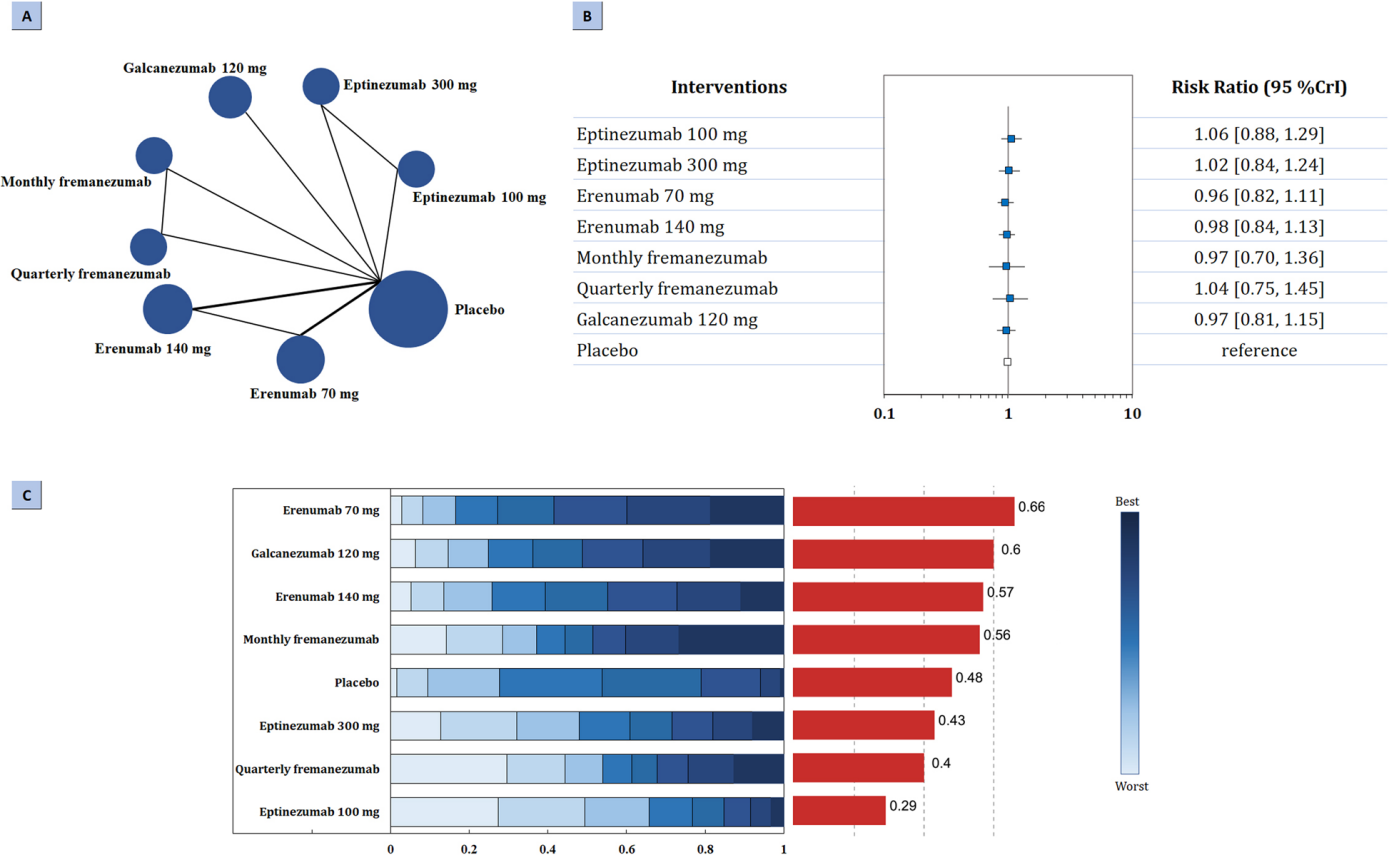
Summary of the primary safety outcome. **A** Network plot of change in TEAEs. The width of the lines is proportional to the number of studies comparing every pair of treatments, and the size of each circle is proportional to the number of participants. **B** The forest plot shows the risk ratio (RR) and credible interval (CrI). **C** Ranking probabilities graph (blue bars) of each treatment agent. The SUCRA values (red bars) for each treatment are as follows: 66% for erenumab 70 mg; 60% galcanezumab 120 mg; 57% erenumab 140 mg; 56% for monthly fremanezumab; 48% for placebo; 43% for eptinezumab 300 mg; 40% for quarterly fremanezumab; 29% for eptinezumab 100 mg. TEAEs: treatment-emergent adverse events; SUCRA: surface under the cumulative ranking curve

Regarding primary safety outcome, the network of comparison of different types of mAbs targeting CGRP was reported in seven trials totaling 2921 participants (Fig. 3A). According to the three-node analysis, neither CGRP mAbs (RR: 0.99, 95% CrI: 0.88 to 1.12) nor CGRP receptor mAbs (RR: 0.99, 95% CrI: 0.87 to 1.13) associated with increased risk of treatment­emergent adverse events (Table 2). Nine-node analysis did not find significant difference. Erenumab 70 mg had the highest probability of rating first to reduce TEAEs (SUCRA 0.66), followed by galcanezumab 120 mg (SUCRA 0.60), erenumab 140 mg (SUCRA 0.57), monthly fremanezumab (SUCRA 0.56), eptinezumab 300 mg (SUCRA 0.43), quarterly fremanezumab (SUCRA 0.40), and eptinezumab 100 mg (SUCRA 0.29). The results were presented in Fig. 3B-C and Fig. A5 in the Supplement.

**Fig. 4:**
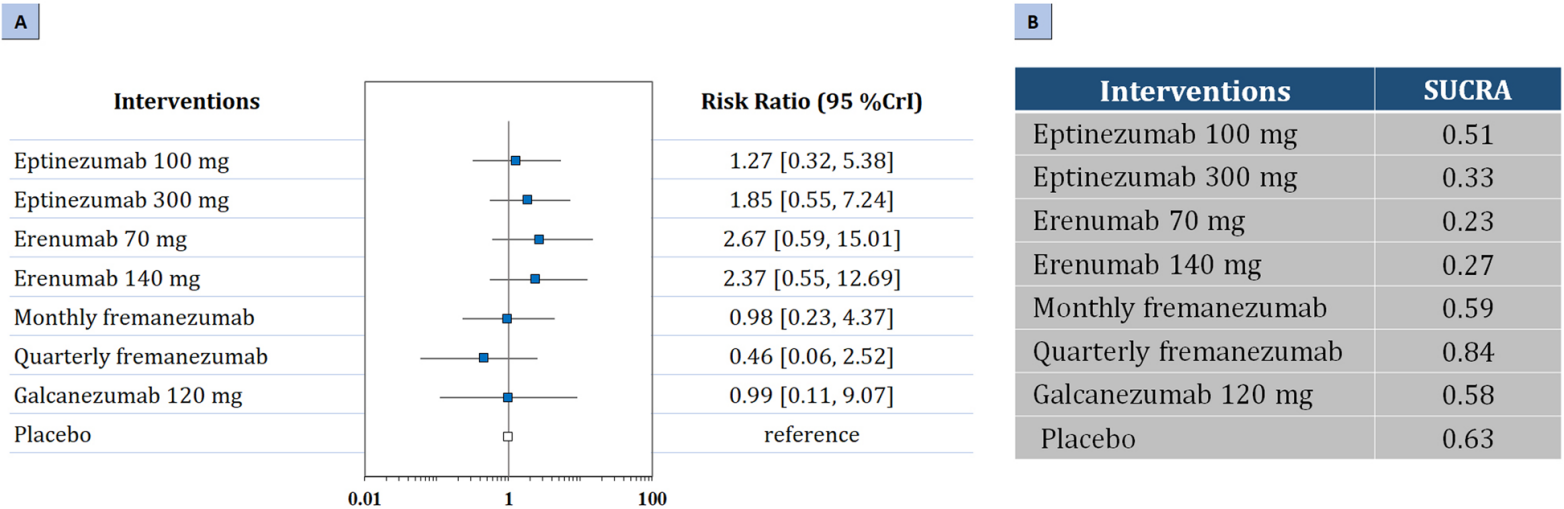
Summary of the secondary safety outcomes. Network plot of (**A**) The forest plot for serious adverse events; (**B**) The SUCRA value of each treatment for serious adverse events. SUCRA: surface under the cumulative ranking curve

Regarding secondary safety outcomes, three-node analysis demonstrated that neither of the treatments increased risk of serious adverse events (Table 2). Nine-node analysis did not show significant difference in any of the comparisons (Fig. 4; Fig. A6 in the Supplement). Quarterly fremanezumab (SUCRA 0.84) had the highest probability of rating first, followed by monthly fremanezumab (SUCRA 0.59), galcanezumab 120 mg (SUCRA 0.58), eptinezumab 100 mg (SUCRA 0.51), eptinezumab 300 mg (SUCRA 0.33), erenumab 140 mg (SUCRA 0.27), and erenumab 70 mg (SUCRA 0.23).

### Risk of Bias and certainty of evidence

Six trials were judged as overall low risk of bias. Only one trial was regarded as unclear risk of bias. Supplementary fig. A7–8 presented the full details of risk of bias assessment for each study. The quality of the evidence for primary outcomes was summarized in Supplementary fig. A9. In general, the certainty of evidence for each agent vs. placebo was judged to be moderate to high.

## Discussion

To our knowledge, this is the first time that different CGRP related mAbs for migraine patients with prior treatment failures have been compared in a network meta-analysis. Our network meta-analysis focused on four CGRP related mAbs involving 3052 patients by pooling data derived from nine RCTs. Pooled results from three-node analysis showed that CGRP mAbs was superior to CGRP receptor mAbs in reducing monthly migraine days and improving at least 50% response rates. Nine-node analysis showed all the treatment agents were similarly efficient in reducing monthly migraine days. Moreover, treatment with fremanezumab or eptinezumab 300 mg provides a significant advantage over erenumab 140 mg in terms of improving at least 50% response rates. All the treatment agents were well tolerated which did not show difference in incidences of TEAEs and serious adverse events in any of the comparisons.

Based on the results of the present analysis, it appears that CGRP mAbs, especially galcanezumab 240 mg, monthly fremanezumab, and eptinezumab 300 mg, seem to be the best choice for the treatment of migraine patients with previous treatment failures. These findings also call for future studies that investigate differences in the efficacy and safety of these drugs in the treatment of migraine in the same patient population.

### Comparison with other studies

This bayesian network meta-analysis compared the relative effects of different mAbs targeting CGRP or its receptor for the treatment of migraine patients with prior treatment failures. In our study we synthesized data across 10 trials to perform three-node and nine-node analysis, and showed the relative ranking of different treatment agents in terms of each outcome.

To the best of our knowledge, most analyses on the same field evaluate the comparative effects of different GCRP related mAbs in patients with migraine regardless of prior medication failure. Therefore, it remains unclear whether these previous findings could be directly applicable to headache problems in the specific population of patients [26, 27]. An additional limitation of previous studies is that most of them used direct method to compare the effectiveness of this medication class with placebo, unable to show the comparative efficacy of different agents in the lack of direct comparison of different types of CGRP related mAbs [28].

### Strengths and limitations of the study

Since network meta-analysis enables different interventions to be evaluated both directly and indirectly even if direct comparison is lacking, this approach has a unique strength over conventional pairwise meta-analysis to provide a more comprehensive analysis of available evidences. A key strength of our study is the use of a network approach to investigate the relative effects of different kinds of CGRP related mAbs for migraine patients with prior treatment failures. By using this method, we were able to compare different therapy agents with placebo both directly and indirectly which give a more precise estimate of the relative efficacy and safety over the pairwise analyses. This method also allowed us to rank the efficacy and safety of different treatment agents. In addition, we reasonably used GRADE tool to judge the quality of evidence for the primary outcomes. These methods are helpful to clinicians in making clinical decisions. Overall, we provided more up-to-date information regarding the reported efficacy and safety of CGRP related mAbs in treating adult migraine patients with prior treatment failures.

There are several limitations need to be noted. First, the disease condition was heterogeneous across the trials, increasing heterogeneity. For example, five trials included patients with episodic migraine, while the others included both episodic and chronic migraine patients. We tried to conduct subgroup analysis based on different group of patients, but the analysis could not be performed due to limited studies. Future studies should report the effects of treatment agents in different types of patient groups.

Second, although galcanezumab 240 mg showed better efficacy in some of the comparisons, we could not evaluate its relative safety with other treatment agents since the original trials did not provide safety data. Future studies should consider this issue and focus on the safety of it in the same patients group.

Third, five trials completed follow-up visit until 24 weeks, while others followed patients up for 12 weeks in a double-blind visit. Given the fact that migraine need treatment for a long time, the dominance of short-term therapy might not be applicable in the actual patients. In fact, evidences from real-world observational studies and open- label extension phase of RCTs confirmed the effectiveness of those drugs.

### Implications in practice

The latest guideline from European Headache Federation (EHF) recommended CGRP related mAbs as a third line treatment for migraine prevention in individuals with migraine [29, 30]. However, in most of the available evidences derived from phase II and phase III RCTs, participants with previous failure of preventive medication classes for migraine were excluded. This implies that efficacy can be different for patients with severe, treatment-resistant migraine. Our findings confirmed the efficacy and tolerability of CGRP related mAbs in the treatment of migraine patients with previous treatment failures. We also suggested CGRP mAbs, especially galcanezumab 240 mg, monthly fremanezumab, and eptinezumab 300 mg, seem to be the best choice for the treatment of these patients. However, cost effectiveness of these novel therapies might vary by intervention, which need to be considered in the same time. Clinicians should consider a comprehensive view based on efficacy, safety, and cost-effectiveness in developing treatment plans.

## Conclusions

In summary, although most agents were well tolerated and showed similar effects, our analysis suggest that galcanezumab 240 mg, monthly fremanezumab, and eptinezumab 300 mg offer the first level in efficacy profile in terms of change in monthly migraine days and 50% response rates in migraine patients with previous treatment failures. These findings are helpful for guideline development and clinicians to make decisions as to which drug to use in the absence of head to-head trials. Future research that investigate differences in the efficacy and safety of these novel agents in the treatment of migraine in the same patient population are needed to validate the present findings.

## Supplementary Information

**Additional file 1: Table A1**. Search terms. **Fig. A1**. Study selection flowchart of randomized controlled trials. **Fig. A2**. League plot of different treatment regimens for the primary efficacy outcome from the network meta-analysis. **Fig. A3**. League plot of different treatment regimens for 50% response rates from the network meta-analysis. **Fig. A4**. League plot of different treatment regimens for 75% response rates from the network meta-analysis. **Fig. A5**. League plot of different treatment regimens for the primary safety outcome from the network meta-analysis. **Fig. A6.** League plot of different treatment regimens for the secondary safety outcome from the network meta-analysis. **Fig. A7.** Risk of bias summary of included trials. **Fig. A8.** Risk of bias graph of included trials. **Fig. A9.** GRADE summary for the primary outcomes.

## Data Availability

All data generated or analysed during this study are included in this published article and its supplementary information files.

## Abbreviations

CGRP: Calcitonin gene-related peptide
RCT: Randomized clinical trials
MMDs: Monthly migraine days
TEAEs: Treatment-emergent adverse events
mAbs: Monoclonal antibodies
PICOS: Patients, intervention, control, outcome, and study
ICHD: International Classification of Headache Disorders
MD: Mean difference
RR: Relative risk
CrIs: Credible intervals
SUCRA: Surface under the cumulative ranking curve
GRADE: Grading of Recommendations Assessment, Development, and Evaluation working group
EHF: European Headache Federation
