# Supplementary material for: Efficacy and safety of monoclonal antibody against calcitonin gene-related peptide or its receptor for migraine patients with prior preventive treatment failure: a network meta-analysis
Source: J Headache Pain. 2022 Sep 8;23(1):105. doi: 10.1186/s10194-022-01472-2 (PMC9454201; doi:10.1186/s10194-022-01472-2)

### Appendix

Table A1: Search terms.

Figure A1: Study selection flowchart of randomized controlled trials

Figure A2: League plot of different treatment regimens for the primary efficacy outcome from the network meta-analysis

Figure A3: League plot of different treatment regimens for 50% response rates from the network meta-analysis

Figure A4: League plot of different treatment regimens for 75% response rates from the network meta-analysis

Figure A5: League plot of different treatment regimens for the primary safety outcome from the network meta-analysis

Figure A6: League plot of different treatment regimens for the secondary safety outcome from the network meta-analysis

Figure A7. Risk of bias summary of included trials.

Figure A8: Risk of bias graph of included trials.

Figure A9: GRADE summary for the primary outcomes.

### Table A1: Search Strategy

| OVID Medline (adapted for other databases) | |
| --- | --- |
| 1 | exp Migraine Disorders/ |
| 2 | (Migraine* OR Migraine Headache* OR Sick Headache* OR Disorder*, Migraine* OR Headache*, Migraine*).ab,kw,ti |
| 3 | 1 or 2 |
| 4 | (eptinezumab OR ALD403).mp |
| 5 | (Galcanezumab OR LY2951742).mp |
| 6 | (erenumab OR AMG334).mp |
| 7 | (fremanezumab OR TEV-48125).mp |
| 8 | 4 or 5 or 6 or 7 |
| 9 | 3 and 8 |
| 10 | exp randomized controlled trial/ |
| 11 | exp controlled clinical trial/ |
| 12 | (randomly OR randomized OR placebo OR trial OR groups).ab,kw,ti |
| 13 | 10 or 11 or 12 |
| 14 | exp animals/ |
| 15 | exp human/ |
| 16 | 14 not 15 |
| 17 | 13 not 16 |
| 18 | 9 and 17 |


### Figure A1: Study selection flowchart of randomized controlled trials

###
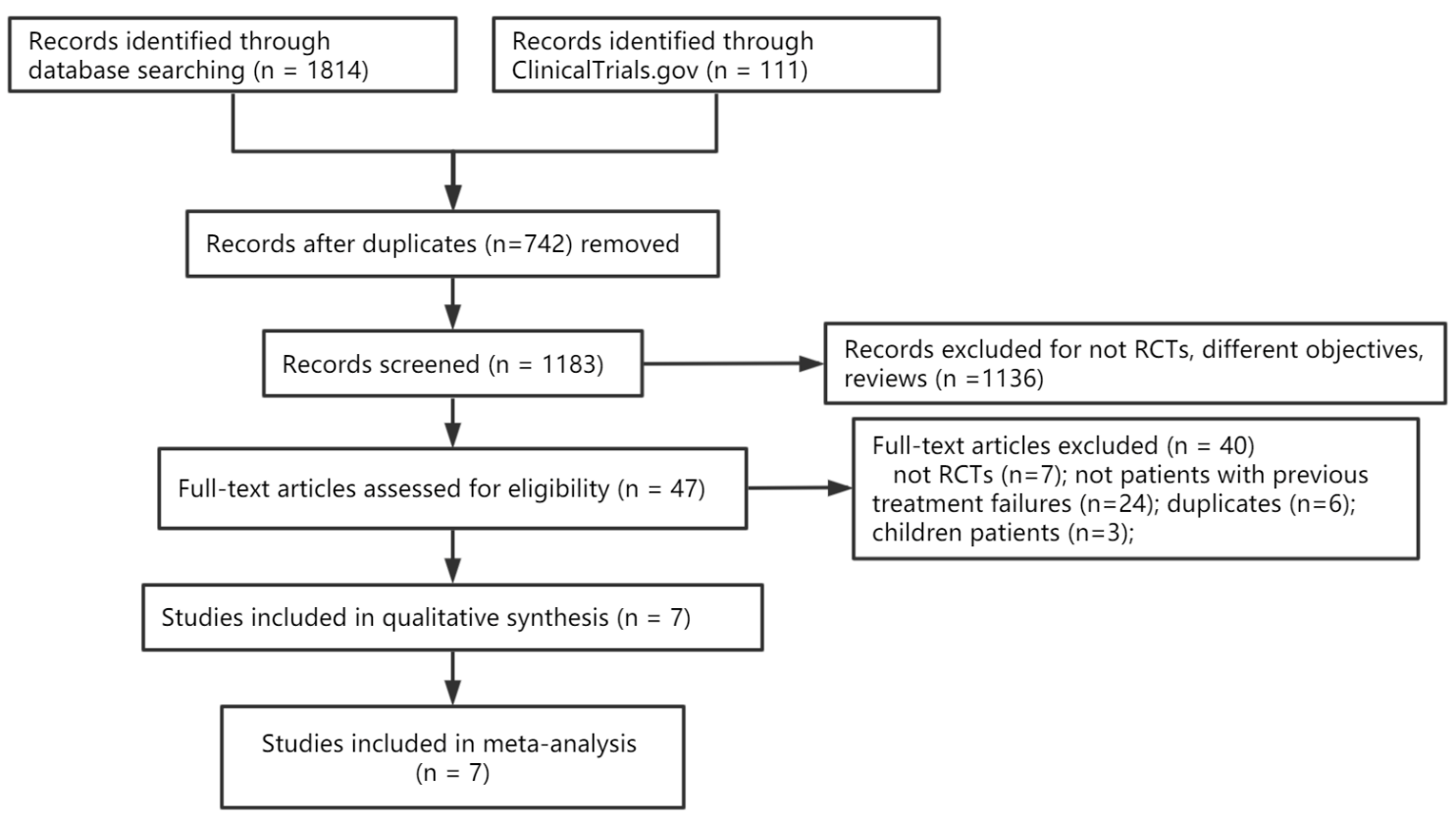


### Figure A2: League plot of different treatment regimens for the primary efficacy outcome from the network meta-analysis


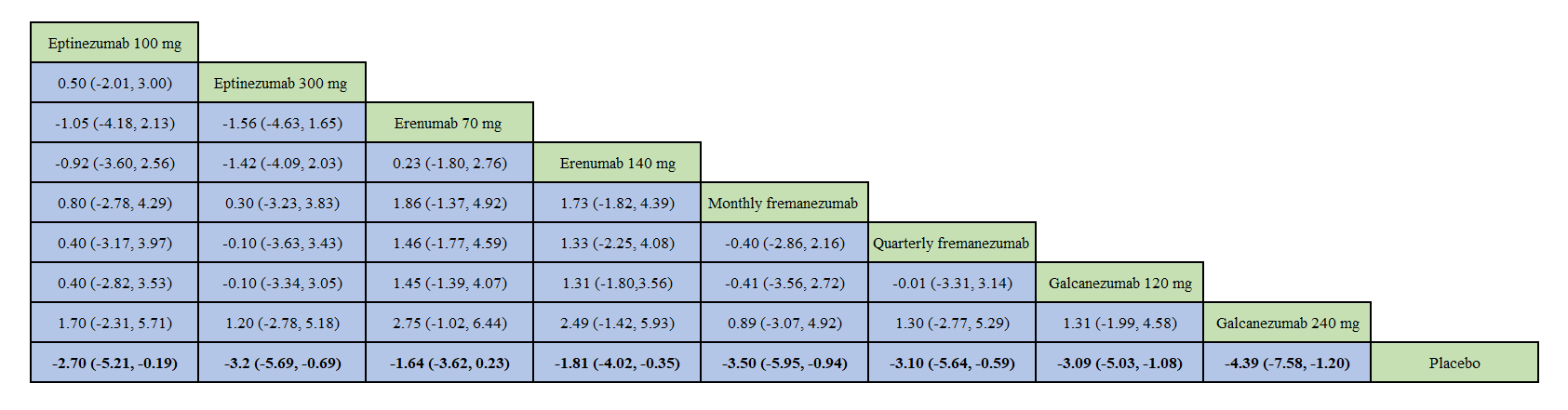


### Figure A3: League plot of different treatment regimens for 50% response rates from the network meta-analysis


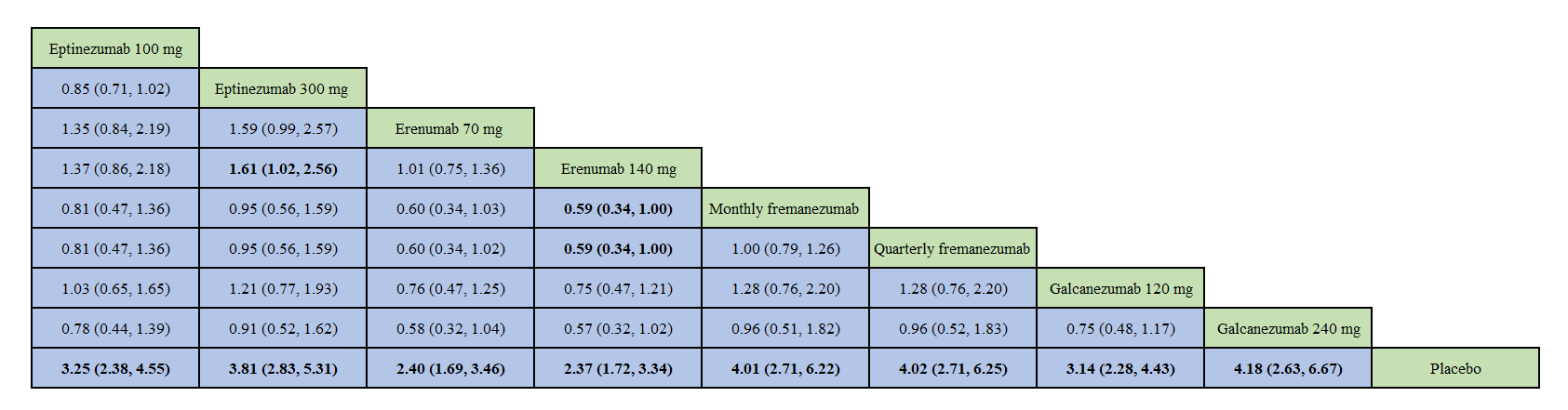


### Figure A4: League plot of different treatment regimens for 75% response rates from the network meta-analysis


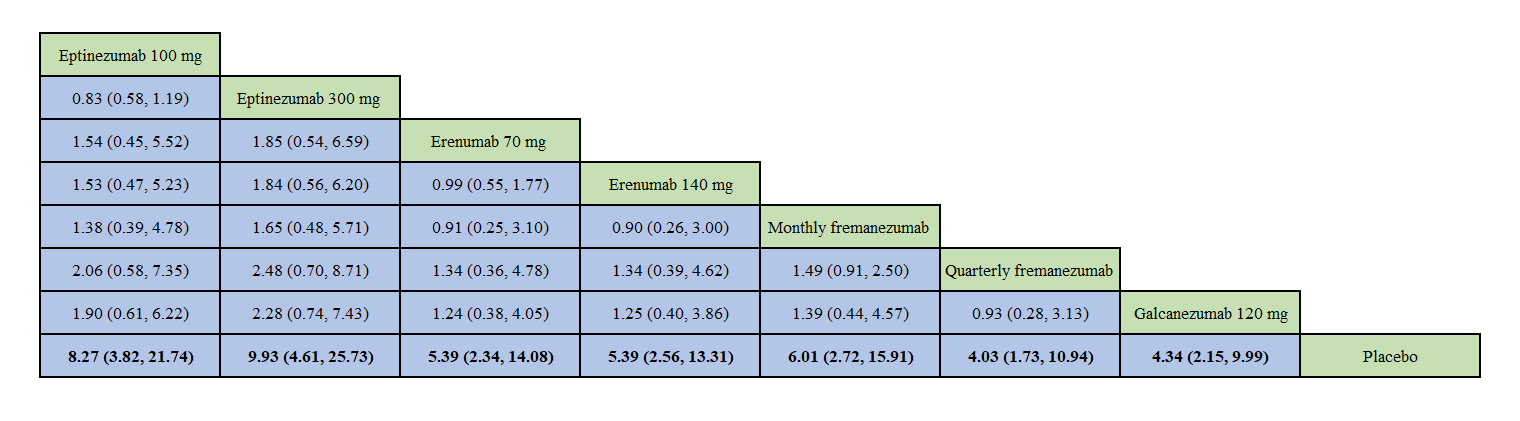


### Figure A5: League plot of different treatment regimens for the primary safety outcome from the network meta-analysis


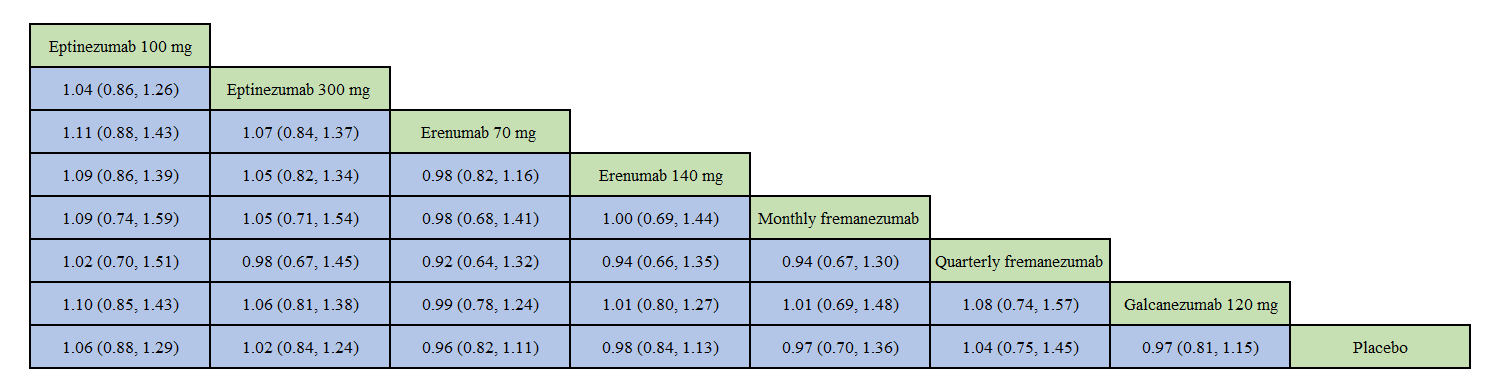


### Figure A6: League plot of different treatment regimens for the secondary safety outcome from nine-node network meta-analysis


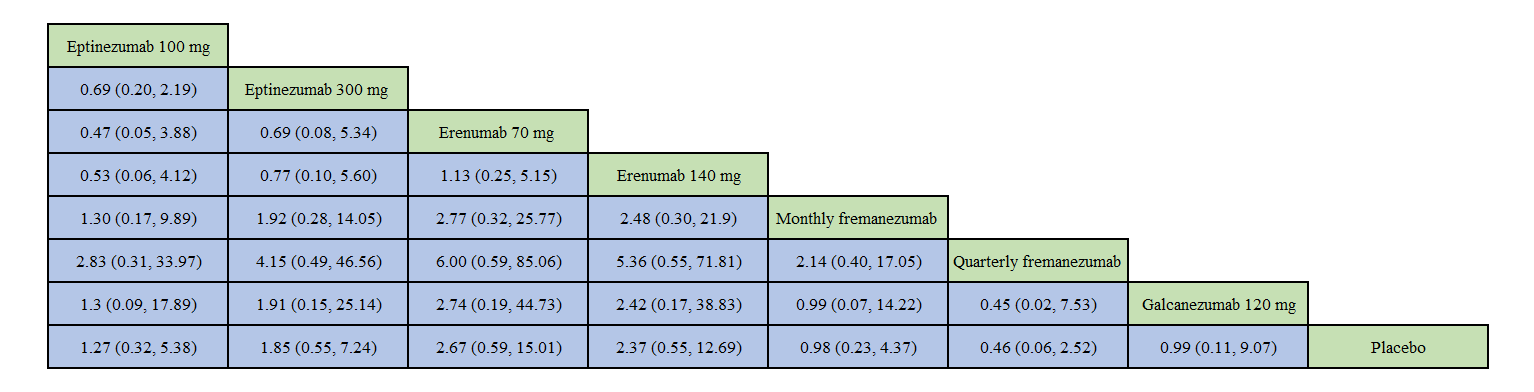


### Figure A7. Risk of bias summary of included trials.


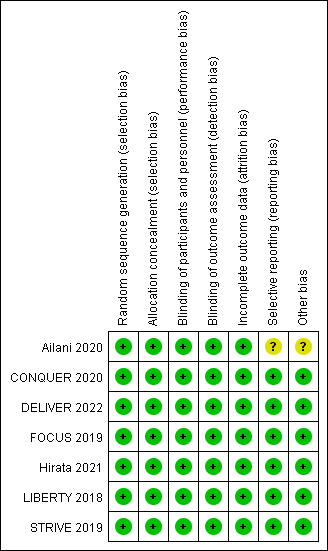


### Figure A8: Risk of bias graph of included trials.

###
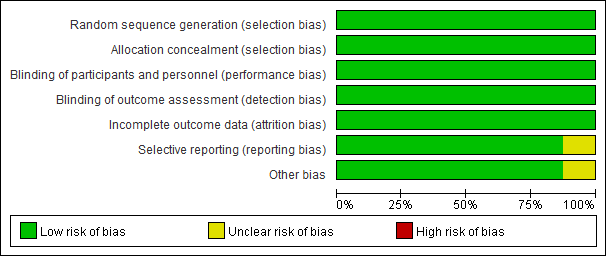


### Figure A9: GRADE summary for the primary outcomes.

###
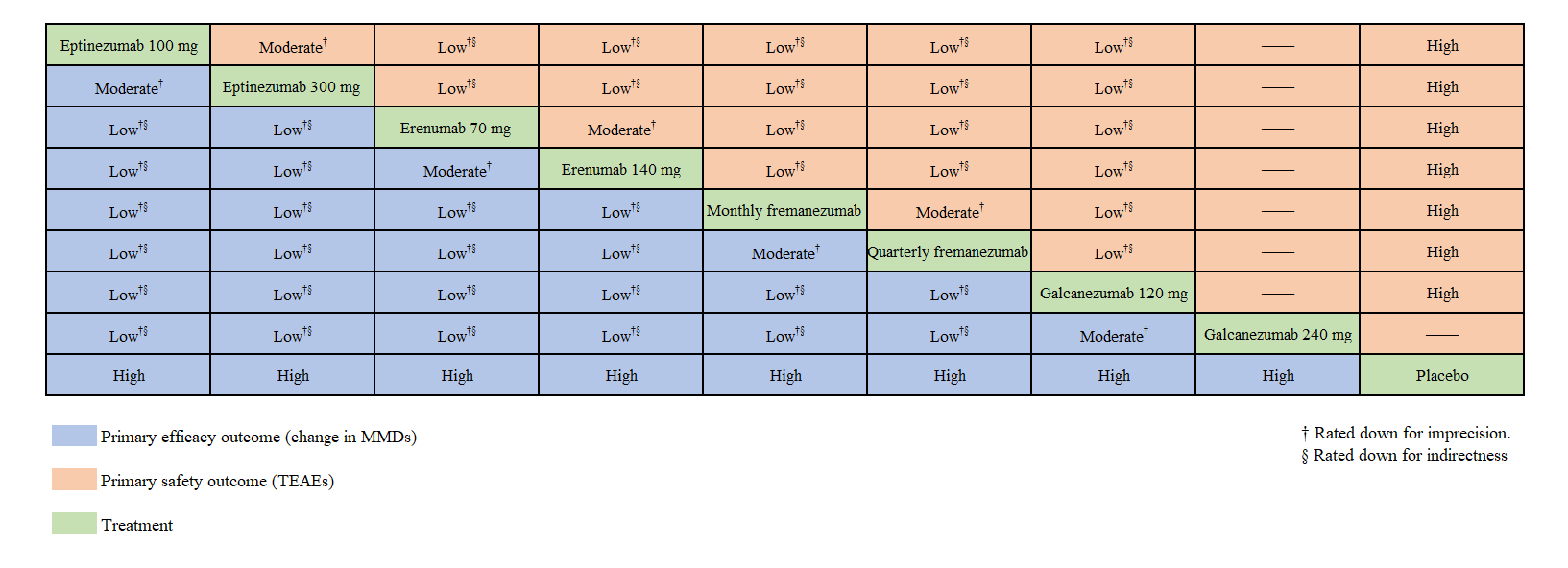

Supplement: Supplementary file 1 — Additional file 1: Table A1. Search terms. Fig. A1. Study selection flowchart of randomized controlled trials. Fig. A2. League plot of different treatment regimens for the primary efficacy outcome from the network meta-analysis. Fig. A3. League plot of different treatment regimens for 50% response rates from the network meta-analysis. Fig. A4. League plot of different treatment regimens for 75% response rates from the network meta-analysis. Fig. A5. League plot of different treatment regimens for the primary safety outcome from the network meta-analysis. Fig. A6. League plot of different treatment regimens for the secondary safety outcome from the network meta-analysis. Fig. A7. Risk of bias summary of included trials. Fig. A8. Risk of bias graph of included trials. Fig. A9. GRADE summary for the primary outcomes. [file 10194_2022_1472_MOESM1_ESM.docx]
